# Cystathionine β-Synthase Mutations: Effect of Mutation Topology on Folding and Activity

**DOI:** 10.1002/humu.21273

**Published:** 2010-05-18

**Authors:** Viktor Kožich, Jitka Sokolová, Veronika Klatovská, Jakub Krijt, Miroslav Janošík, Karel Jelínek, Jan P Kraus, David N Cooper

**Affiliations:** 1 First Faculty of Medicine, Charles University in Prague and General University Hospital in Prague, Institute of Inherited Metabolic DisordersPrague, Czech Republic; 2 Faculty of Science, Charles University in Prague, Department of Physical and Macromolecular ChemistryPrague, Czech Republic; 3 University of Colorado School of Medicine, Department of PediatricsAurora, Colorado

**Keywords:** homocysteine, cystathionine β-synthase deficiency, CBS, homocystinuria, folding, misfolding, topology, *E. coli*

## Abstract

Misfolding of mutant enzymes may play an important role in the pathogenesis of cystathionine β-synthase (CBS) deficiency. We examined properties of a series of 27 mutant variants, which together represent 70% of known alleles observed in patients with homocystinuria due to CBS deficiency. The median amount of SDS-soluble mutant CBS polypeptides in the pellet after centrifugation of bacterial extracts was increased by 50% compared to the wild type. Moreover, mutants formed on average only 12% of tetramers and their median activity reached only 3% of the wild-type enzyme. In contrast to the wild-type CBS about half of mutants were not activated by S-adenosylmethionine. Expression at 18°C substantially increased the activity of five mutants in parallel with increasing the amounts of tetramers. We further analyzed the role of solvent accessibility of mutants as a determinant of their folding and activity. Buried mutations formed on average less tetramers and exhibited 23 times lower activity than the solvent exposed mutations. In summary, our results show that topology of mutations predicts in part the behavior of mutant CBS, and that misfolding may be an important and frequent pathogenic mechanism in CBS deficiency. Hum Mutat 31:1–11, 2010. © 2010 Wiley-Liss, Inc.

## Introduction

Cystathionine β-synthase (CBS) deficiency (MIM# 236200, also known as homocystinuria due to CBS deficiency) is a well-known multisystemic inborn error of metabolism with widely varying estimates of frequency. Although the number of patients with clinically ascertained CBS deficiency is rather low and the prevalence is only 1:330,000 worldwide [Mudd et al., 2001], the molecular epidemiological studies suggest an incidence of around 1:10,000 in several European populations [Gaustadnes et al., 1999; Janosik et al., 2009; Refsum et al., 2004]. Classical form of this disease manifests by combination of neurological, connective tissue, and vascular involvement in childhood, whereas milder form of the disease may be diagnosed later in adulthood with only vascular manifestation. Severity of the untreated disease is determined mostly by the underlying genetic defect, and patients with milder forms of CBS deficiency usually respond to pharmacological doses of pyridoxine by a marked decrease of their total homocysteine plasma levels.

More than 150 mutant alleles have been described in patients with CBS deficiency with a representation of all types of genetic variants (http://www.uchsc.edu/cbs/cbsdata/cbsmain.htm). Mis-sense mutations are the most common variants described in the CBS gene, representing 87% of the 753 analyzed patient-derived CBS alleles as of April 2010. In a previous study we have proposed that missense CBS mutant enzymes may misfold, and that this behavior may be responsible for their pathogenicity [Janosik et al., 2001b]. This observation was later confirmed by other groups and also by expressing mutants in systems other than *Escherichia coli* (for references, see Table 1). The finding that misfolding and decreased activities of some mutant proteins may be corrected with the help of chemical chaperones further supports the role of misfolding in the pathogenesis of CBS deficiency [Singh et al., 2007]. Despite many years of research novel data on structural and biochemical properties of CBS mutant proteins are scattered in the literature, and a comprehensive analysis of the folding-related findings is missing.

**Table 1:** Panel of Studied Mutations

	Mutation		Solvent accesibility				
			
Location	Protein change	cDNA change	Absolute (A^2^)	Relative (%)	Class	Number of alleles	Properties of mutant enzymes
Active site	p.G148R	c.442G>A	1.31	1.80	B	3	ECE: tetramer, 0–5.82%WT activity [Katsushima et al., 2006; Orendac et al., 2004; Urreizti et al., 2006]
	p.G305R	c.913G>A	3.58	4.90	B	1	
	p.G307S	c.919G>A	0.16	0.50	B	88	ECE: undetectable activity [Hu et al., 1993]; SCE: <1.7% WT activity [Kruger et al., 2003]
Heme binding pocket	p.H65R	c.194A>G	22.01	30.30	S	3	PE: 11.5% WT activity, 75% PLP saturation, 40% heme saturation [Ojha et al., 2002]; ECE: undetectable activity [Chen et al., 1999]
	p.T262R	c.785C>G	0.14	0.20	B	1	
	p.R266K	c.797G>A	1.76	2.90	B	10	PE: *V*_max_ 28–114% of WT, *K*_*m*[PLP]_: 3 × of WT; SCE: tetramer, 49%WT activity, *K*_*m* [PLP]_: 10 × of WT [Chen et al., 2006]
Other locations in active core	p.E144K	c.430G>A	0.10	0.20	B	12	ECE: <1%WT activity [Dawson et al., 1997; Janosik et al., 2001b]
	p.C165Y	c.494G>A	1.85	3.00	B	8	ECE: 0–3%WT activity [Gordon et al., 1998; Kluijtmans et al., 1998, 1999]
	p.N228K	c.684C>G/A	0.06	0.10	B	4	ECE: aggregates, 0% WT activity [Gaustadnes et al., 2002]
	p.I278T	c.833T>C	0.00	0.00	B	146	PE: *V*_max_ 0.8–1.4% of WT, *K*_*m* [PLP]_: 1.4 × of WT [Chen et al., 2006]; ECE: aggregates, 0–2%WT activity [Janosik et al., 2001b; Kluijtmans et al., 1998, 1999; Kozich and Kraus, 1992]; SCE: WTactivity, tetramer, <2–5% WT activity, *K*_*m* [PLP]_: 0.7 × of WT [Chen et al., 2006; Kruger et al., 2003; Shan et al., 2001; Singh et al., 2007]; MLE: ∼2% WT activity [Wang et al., 2005]
	p.P49L	c.146C>T	35.01	61.90	S	3	
	p.K102N	c.306G>C	17.74	43.40	S	1	ECE: 21–34%WT activity [de Franchis et al., 1994]
	p.R125Q	c.374G>A	8.57	16.60	S	13	ECE: 0–1.9%WT activity [Marble et al., 1994; Sebastio et al., 1995]
	p.T191M	c.572C>T	6.93	11.20	S	137	ECE: 1%WT activity [Kluijtmans et al., 1998]
	p.E302K	c.904G>A	15.03	24.50	S	6	ECE: shorter product -partially degradaded, 4.9% WT activity [de Franchis et al., 1999]
	p.R369C	c.1105C>T	4.55	10.90	S	4	SCE: does not affect CBS function [Kim et al., 1997]; ECE: 34% WT activity [Janosik et al., 2009]; CHO: 54% WT activity, *V*_max_ 29% of WT, *K*_*m* [serine]_: 0.8 × of WT, *K*_m_[*homocysteine*]: 1.3 × of WT [Janosik et al., 2009]
Dimer-dimer interface	p.P78R	c.233C>G	47.79	65.80	S	2	ECE: 13–61%WT activity [de Franchis et al., 1994]
	p.A114V	c.341C>T	8.94	18.07	S	10	ECE: tetramer + aggregates [Janosik et al., 2001b], 46.3–50%WT activity [de Franchis et al., 1999; Sebastio et al., 1995]
	p.E176K	c.526G>A	16.35	26.60	S	2	ECE: aggregates, 0% WT activity [Janosik et al., 2001b]
	p.V180A	c.539T>C	5.15	15.50	S	1	ECE: 3–8% WT activity [Kluijtmans et al., 1998, 1999]
Connection loop between active core and regulatory domain	p.W409_ G453del	r.1224_1358del			B	19	ECE: aggregates [Janosik et al., 2001b], inactive [Kozich and Kraus, 1992]
Regulatory domain-first CBS domain	p.P422L	c.1265C>T	4.89	8.70	B	1	ECE: ∼160% WT activity, 0.16 × activation by AdoMet compared to WT;CHFE: ∼150% WT activity [Maclean et al., 2002]
	p.I435T	c.1304T>C	16.11	38.50	S	1	ECE: tetramer [Janosik et al., 2001a], ∼290% WT activity, 0.03 × activation by AdoMet compared to WT [Maclean et al., 2002]; CHFE: ∼100% WT activity [Maclean et al., 2002]
	p.R439Q	c.1316G>A	36.79	71.20	S	3	ECE: 30%WT activity [Dawson et al., 1997]
	p.D444N	c.1330G>A	18.73	45.80	S	13	PE: 1.5 × of WTactivity, *K*_act[AdoMet]_ 62 × of WT [Evande et al., 2002]; ECE: 96–100% WT activity [Kluijtmans et al., 1998, 1999]
	p.S466L	c.1397C>T	0.23	0.40	B	1	ECE: ∼310% WT activity, 0.05 × activation by AdoMet compared to WT [Maclean et al., 2002]; CHFE: ∼300% WT acivity [Maclean et al., 2002]; MLE: tetramer, ∼400% WT activity [Gupta et al., 2008]
Regulatory domain-second CBS domain	p.L539S	c.1616T>C	1.93	5.70	B	2	

Mutations selected for this study are grouped according to their location in different domains of the CBS three-dimensional structure [Meier et al., 2001, 2003]. Nucleotide numbering reflects the cDNA numbering with +1 corresponding to the A of the ATG translation initiation codon in the reference GenBank sequence NM_000071.2, according to the Journal guidelines (http://www.hgvs.org/mutnomen). The initiation codon is codon number 1. Solvent accessibility has been calculated using the program Modeller, mutations are classified based on predicted solvent accessibility as solvent exposed (S, accessible surface area larger than 40 A^2^ and/or relative accessible surface area larger than 9%) or buried (B). Number of alleles carrying particular mutation has been taken from the CBS mutation database (http://www.uchsc.edu/cbs/cbsdata/cbsmain.htm) as of April 7, 2010. Data on properties of mutant enzymes has been extracted from literature, expression systems are abbreviated as follows: ECE, *E. coli* crude extracts; SCE, *S. cerevisiae* crude extracts; CHFE, Chinese hamster fibroblasts crude extracts; CHO, Chinese hamster ovary cells crude extract; MLE, mouse liver extracts; PE, purified enzymes.

In this study, we examined systematically catalytic and folding-related structural properties of a panel of 27 mutants located in different functional domains of the CBS molecule. The panel of mutations contained, among others, the most prevalent mutant CBS alleles to well represent the molecular mechanisms relevant for the majority of patients suffering from CBS deficiency. Because purification of CBS mutant proteins for further studies is not universally achievable, we decided to study properties of mutant proteins in crude *E. coli* extracts, thus obviating any possible loss of mutants during the purification procedure. We studied the solubility of the mutant proteins in sodium dodecyl sulfate (SDS), their oligomeric structure, activity, response to S-adenosylmethionine (AdoMet) and S-adenosylho-mocysteine (AdoHcy), and changes of these parameters after expression at a decreased temperature. We subsequently examined whether the location of mutant residues in the three-dimensional CBS structure in respect to solvent accessibility is a determinant of their properties. We show that mutations of residues buried in protein globule appear to have more severe effect on folding than those exposed to the solvent.

## Methods

### Nucleotide and Codon Numbering

Nucleotide numbering reflects the cDNA numbering with +1 corresponding to the A of the ATG translation initiation codon in the reference GenBank sequence NM_000071.2, according to the Journal guidelines (http://www.hgvs.org/mutnomen). The initiation codon is codon number 1.

### Molecular Dynamics Simulation

In silico analyses presented in this article can be divided in two parts. The first part presents an analysis of mutant structures of catalytic domain of CBS, and the second part presents analysis of CBS regulatory domain. X-ray structure of the active core of the CBS protein (PDB code 1JBQ) [Meier et al., 2001] was utilized as the starting model for the catalytic domain. 1JBQ X-ray structure contains the first 413 residues of the protein and covers the whole catalytic domain. First, we optimized the native structure in program Modeller (http://salilab.org/modeller) [Sali and Blundell, 1993], which combines empirical spatial restraints and a CHARMM force field into an objective function and employs conjugate gradients and molecular dynamics with simulated annealing methods for energy optimization. Model refinement level property of the Modeller procedure was set to very low. In the next step the optimized native structure was mutated in silico, and mutant structures were optimized in the same way.

Although a model of the regulatory domain of human CBS (residues 415–543) has been published a few years ago [Sen et al., 2005] the X-ray structure of this part of the CBS molecule is as yet unknown. We generated our own model of this portion of the wild-type CBS using the comparative structure modeling. As the initial model we used X-ray structure of the conserved CBS domain of the protein TA0289 from *Thermoplasma acidophilum* (PDB code 1PVM) [Proudfoot et al., 2008]. Sequence of the CBS regulatory domain was aligned with the TA0289 sequence and coordinates of its native structure were derived from the 1PVM coordinates (data not shown). The native structure was then optimized and mutant structures were computed as in case of the active core. For the native as well as all computed mutated structures of both domains, the surface accessible area of amino acid residues was calculated and normalized by maximum accessible surface area for the corresponding residue type by the program Modeller [Sali and Overington, 1994]. A residue was considered exposed if its accessible surface area was larger than 40 A2 and/or if its relative accessible surface was larger than 9%, while considered buried otherwise [Mirkovic et al., 2004].

### Expression in *E. coli*

The wild-type CBS and mutant constructs were derived from the pHCS3 expression plasmid [Kozich and Kraus, 1992]. Seven mutant plasmids, namely, the c.341C>T (p.A114V), c.442G>A (p.G148R), c.526G>A (p.E176K), r.1224_1358del; p.W409_G453del, c.1265C>T (p. P422L), C.1304T>C (p.I435T), and c.1397C>T (p.S466L) were prepared using the previously described procedure of replacing a fragment of wild-type CBS expression plasmid with the equivalent restriction fragment derived from the mutant patient-derived cDNA [Kozich and Kraus, 1992]. The remaining mutations were introduced into the wild-type expression plasmid by help of the GeneTailor site-directed mutagenesis kit (Invitrogen, Carlsbad, CA) according to the manufacturer's procedure. The sequences of mutagenic primers are given in Supp. Table S1. The authenticity of all constructs was verified by dideoxy sequencing using ALF sequencer (Amersham Pharmacia Biotech, Piscataway, NJ).

The expression plasmids were used to transform *E. coli* DH5α cells (Gibco BRL, Carlsbad, CA). The cells were grown at 37 or 18°C in SOB (Super Optimal Broth) media with 100μg/ml ampicilin to OD_600_ of ∼0.5, expression of CBS was carried out in the presence of isopropyl β-D-1-thiogalactopyranoside (final concentration 0.33 mmol/l) at 37 or 18°C for 3 hr or overnight, respectively. Each mutant protein was expressed in at least five independent experiments on separate days (i.e., three and two expression at 37 and 18°C, respectively). Each series of mutant proteins contained also bacteria transformed with the pKK388.1 vector lacking the CBS insert as a negative control, and the wild-type CBS that was used to normalize data.

Lysates from *E. coli* cell pellets were prepared by sonication in lysis buffer (50 mmol/l Tris-Cl pH 8, 10 mmol/l EDTA, 1 mmol/l DTT, 0.1% lysozyme) with the addition of commercial Protease Inhibitor Cocktail (Sigma Aldrich, St. Louis, MO) according to manufacturer's protocol. A 50-μL aliquot of each sonicate was saved for the analysis of CBS in the particulate and nonparticulate fraction, the remaining sonicate was centrifuged at 15,000 × *g* for 30 min at 4°C, and the supernatant was aliquoted and stored for subsequent analyses at −85°C. Protein concentration in the supernatant was determined by the method of Lowry with bovine serum albumin as a standard [Lowry et al., 1951].

### Optimizing Extraction of Aggregated CBS

We tested several methods that have been previously used by other authors to extract aggregated proteins, such as amyloid, from bacterial lysates [Carra et al., 2005]. Optimization was carried out with the wild-type CBS and four mutant enzymes (p.A114V, p.V180A, p.K102N, and p.I278T). We have compared the effects of the following denaturing reagents: (1) 1.5% SDS in the presence of 0.1 M b-mercaptoethanol (without preincubation), (2) 3% SDS in the presence of 0.1 M β-mercaptoethanol (without preincubation), (3) 6 M urea, 1.5% SDS, and 0.1 M β-mercaptoethanol (1hr incubation at room temperature), (4) 6 M urea/thiourea (v/v 2:1) containing 1.5% SDS and 0.1 M b-mercaptoethanol (1hr incubation at room temperature), and (5) 100% formic acid (30 min incubation at 37°C) followed by a lyophilization and dissolution in denaturating solution containing 1.5% SDS and 0.1 M β-mercaptoethanol. After incubation of uncentrifuged sonicates with different denaturing reagents for the indicated time the samples were boiled for 10 min at 100°C and analyzed by SDS-PAGE and Western blotting as described below. These experiments revealed, that methods (2) and (5) provided a higher yield of immunoreactive CBS signal compared to the other methods. Owing to a simplicity and a sufficient efficacy of method (2), we subsequently used the extraction of insoluble CBS from pelets and supernatants by 3% SDS.

### Analysis of Particulate and Nonparticulate Fraction

Fifty microliters of sonicated cell extracts were centrifuged at 4°C at 15,000 × *g* for 30 min, the supernatants were saved and the pellets were resuspended in 50μL of denaturation buffer (50 μmmol/l Tris-Cl pH8, 0.1 mol/l β-mercaptoethanol, 3% SDS). To solubilize the CBS present in the samples 5 μL of either the resuspended particulate fraction or of the supernatant were mixed with 15 μL of the denaturation buffer and boiled at 100°C for 10 min; subsequently, 4 μL of electrophoresis loading buffer supplemented with 0.1 mmol/l dithiothreitol were added and the solution was heated for 10min at 100° prior to loading on the SDS-PAGE gel. Samples of the supernatant and particulate fraction of each mutant protein were loaded next to each other on the gel and subjected to electrophoresis and Western blot analysis (see below the section Electrophoresis and Western Blot Analysis); each gel also contained the supernatant and particulate fraction of a negative control and of bacteria expressing wild-type CBS. The sharply demarcated signal of CBS in each fraction was quantified by chemiluminescence after subtracting the background of the negative control. For each mutant the signal in the supernatant and the particulate fractions were added, thus yielding the total CBS antigen of the respective mutant. This total signal was subsequently normalized to the total signal of wild-type CBS enzyme after correction for the protein concentration.

### Electrophoresis and Western Blot Analysis

Separation of CBS under denaturing conditions was carried out in 4–15% gradient native polyacrylamide gels (Criterion Precast Gel, BioRad, Hercules, CA) with standard Laemmli SDS-containing buffer system [Laemmli, 1970]. For the analysis of CBS under native conditions cell lysates containing 10 μg of total protein were separated using the same precast polyacrylamide gels with standard Laemmli buffer system without SDS [Laemmli, 1970].

Proteins separated by either SDS-PAGE or native PAGE were transferred onto polyvinylidene difluoride membrane (Immobilon-P, Millipore, Billerica, MA) using semidry blotting transfer technique. After transfer nonspecific binding sites were blocked by overnight incubation with 5% nonfat dry milk in 1 × phosphate-buffered saline solution (PBS), pH 7.0, and 0.2% Tween 20. The membrane was subsequently incubated with immunopurified anti-CBS antibody diluted 1:5,000 in 1 × PBS containing 3% bovine serum albumin for 1 hr and after a series of washes with secondary antirabbit IgG antibody conjugates with HRP (Pierce, Rockford, Il) for 30min. The secondary antibody was diluted 1:30,000 in 1 × PBS/0.2% Tween 20 containing 5% nonfat dry milk. After a second series of washes the signal was visualized using the West Pico Super Signal system (Pierce Biotechnology), followed by bioimaging system ChemiGenius-Q (Syngene Inc., Federick, MD) with a cooled CCD camera. The amount of sharply demarcated fractions of tetramers and oligomers and of fuzzy high-molecular weight aggregates were quantified with Gene Tools software (Syngene Inc.) after the subtraction of background on an *E. coli* extract lacking CBS that was analyzed in each series.

### Estimation of Molecular Weight by Ferguson Plot

Molecular weight of CBS fractions with varying mobility was estimated using a modification of the method published by Ferguson [Ferguson, 1964; Hedrickand Smith, 1968]. The original method is based on electrophoresing the analysed protein together with markers in a series of polyacrylamide gels with increasing concentration of acrylamide. We have determined the relative mobilities normalised to the dye front (*R_f_*) of commercial molecular mass markers (Blue Range, Pierce) ferritin (440kDa), catalase (232 kDa), urease (272 and 545 kDa), bovine serum albumin (BSA) (66 and 132 kDa), and egg albumin (45 kDa) using 10-cm long 5, 6, 7, 8, 9, and 10% native polyacrylamide gels and Laemmli electophoresis buffers without SDS [Laemmli, 1970] after staining of the gels by Coomassie blue R The *E. coli* extracts containing mutant and wild-type CBS were separated by the same series of polyacrylamide gels, the CBS fractions were visualized by Western blotting and immunodetection as described above, and the relative mobilities normalizes to the dye front have been determined.

The logarithms *R_f_* of each band of the standard proteins were plotted against the acrylamide gel concentrations, and the slope of regression line of each standard protein was calculated. The calibration line was derived as a function of the logarithms of negative slopes and the molecular weights of the standard. The negative slopes of all oligomeric forms of wild-type CBS and of selected mutant proteins were determined by the same method, and these values were used for calculation of molecular weight of the different CBS fractions. The bacterial extracts used for this analysis contained the wild-type CBS, the p.K102N, p.S466L, p.E302K, p.G305R, p.G307S, and p.R125Q variant proteins expressed at 18°C.

### CBS Enzymatic Assay

The CBS activity in *E. coli* lysates was assayed by modification of the previously published procedure [Kozich and Kraus, 1992]. The assay mixture contained 100 mM Tris-HCl (pH 8.6), 0.5 mg/ml BSA, 0.5 mM pyridoxal 5′-phosphate, 1 mM dithiothreitol, 10 mM homocysteine, 10 mM 2,3,3-^2^H-labeled serine (Cambridge Isotope Laboratories) and 27.5 ml of *E. coli* extract in lysis buffer containing 75 μg of bacterial protein. The assay mixture was incubated at 37°C for 1 hr in a total volume of 50 μl. For measurement of responsivity to adenosyl compounds the assay mixture contained either 0.5 mM AdoMet or AdoHcy. The activity was determined by the LC-MS/MS measurement of the product of enzyme reaction, 3,3-^2^H-labeled cystathionine, using the commercially available kit for amino acid analysis (EZ:faast, Phenomenex, Torrance, CA) (Krijt et al., unpublished).

### Statistical Analyses

Means, standard deviations, and medians were determined using the spreadsheet Microsof Office Excel 2003 (Microsoft, Czech Republic), linear regression analysis was carried out using the same software.

## Results

### Selection of Mutations and In Silico Analysis

**Fig. 1:**
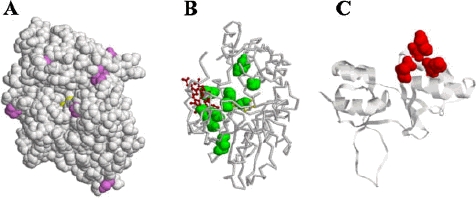
Location of studied mutations in three-dimensional CBS structure. **A**: Mutations located at the surface of the active core are shown in magenta, pyridoxal 5′-phosphate (PLP) is shown in yellow. **B**: Mutations buried within the active core are indicated in green, heme and PLP molecules are shown in red and yellow, respectively. **C**: Mutations located at the surface of the carboxyterminal regulatory CBS domain are shown in red.

To gain clinically as well as mechanistically relevant insight into the role of misfolding in CBS deficiency we first selected a series of naturally occurring CBS mutations for further laboratory studies. The set of nine most prevalent mutations having a frequency of at least 10 alleles in the CBS Mutation Database was selected; these included the p.A114V, p.R125Q, p.E144K, p.T191M, p.R266K, p.I278T, p.G307S, p.W409_G453del, and p.D444N. This set was expanded by additional 18 less frequent mutations known to be localized in different domains of the CBS protein [Meier et al., 2001, 2003]. For the list of mutations see Table 1; location of the mutations in the CBS active core and in the modeled carboxyterminal domain is shown in Figure 1. The final series reflects the molecular pathology of CBS deficiency as these 27 mutations are present on about 70% of all pathogenic CBS alleles known in humans to date.

Based on our previous work [Singh et al., 2007], we hypothesized that CBS mutations of residues buried in protein globule may have more severe effect on folding than those exposed to the solvent. After selecting the mutants we performed an in silico analysis with calculation of the solvent-accessible surface area. Based on previously published criteria we considered mutant residues with relative and absolute solvent-accessible surface area higher than 9% and/or 40 A^2^, respectively, as being solvent exposed while the rest of the mutations were considered being buried in the globule [Mirkovic et al., 2004]. Table 1 shows the predicted solvent accessibility and demonstrates that mutations belonging to these two classes are represented almost equally in the series of 27 variant proteins.

### Total Amount of CBS Mutant Proteins Expressed in *E. coli* and Their Solubility

It has been shown previously that some CBS mutant proteins expressed in bacteria misassemble and aggregate [de Franchis et al., 1999; Gaustadnes et al., 2002; Janosik et al., 2001b; Singh et al., 2007; Yamanishi et al., 2006]. It is conceivable that aggregated mutant CBS polypeptides will form inclusion bodies, and that a substantial fraction of misfolded CBS proteins may thus escape detection if the commonly used supernatant fraction of bacterial extracts is utilized for Western blotting analysis.

To test this assumption we first optimized a method for extracting water-soluble as well as aggregated and water-insoluble CBS from bacteria expressing wild -ype and four mutant enzymes (for details, see the Methods section). Boiling in 3% SDS was subsequently used for the entire series of 27 mutant proteins to determine the amount of CBS antigen in both the particulate (i.e., pellet after centrifugation) and nonparticulate (i.e., supernatant) fractions of sonicated bacterial extracts. The sum of CBS signal in both fractions is referred to as the total SDS-soluble CBS antigen.

**Figure 2:**
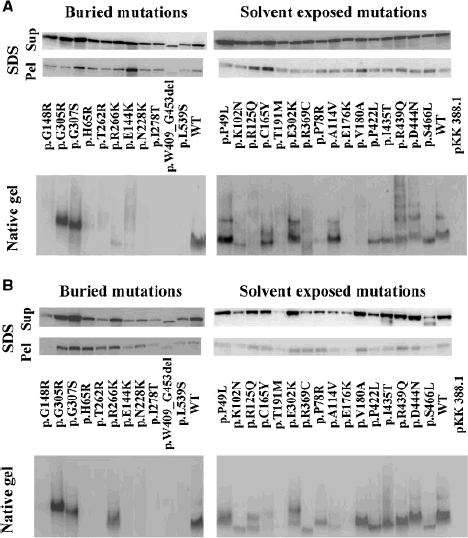
Amounts of mutant CBS antigen in fractions of bacterial extracts. **A**: Mutant proteins expressed at 37°C; **B**: Results of expression of mutant proteins at 18°C. Top part of panels. The amount of SDS-soluble CBS antigen in particulate (Pel, pellet) and nonparticulate (Sup, supernatant) fractions of bacterial extracts obtained by centrifugation was determined by SDS-PAGE and Western blotting as described in the Methods section. The blots in this figure are only shown to demonstrate the presence of CBS antigen in both fractions and do not reliably reflect the proportion between the fractions; the particulate fractions and the supernatant fractions were loaded on separate gels, whereas in the experimental blots used for quantification these two fractions of each mutant were loaded next to each other on the same gel (data not shown). Only a single representative sample from two to three independent expression experiments has been loaded for this publication gel. Bottom part of panels. The quaternary structure of CBS mutants was assessed in the water-soluble nonparticulate fraction of bacterial extracts by electrophoresis under native conditions followed by Western blotting; sharply demarcated fractions are tetramers and higher oligomers. Only a single representative sample from two to three independent expression experiments has been loaded for this publication gel. Solvent exposed mutations, mutations with accessible surface area larger than 40 A^2^ and/or with relative accessible surface larger than 9% (with the exception of mutations p.C165Y, p.P422L, and p.S466L that belong to buried mutations) buried mutations, remaining mutations from the panel are grouped here (with the exception of mutation p.H65R that belong to solvent exposed mutations). WT, bacterial extracts containing wild-type CBS; pKK 388.1, extracts of bacteria transformed with the empty pKK 388.1 plasmid lacking any CBS.

Using the SDS extraction we observed that all 27 mutant proteins were present in detectable amounts not only in the supernatants but more importantly also in the particulate fractions of the bacterial extracts. The presence of CBS antigen in both fractions is shown in Figure 2; however, this publication gel does not allow inferring either on relative proportions of the mutant proteins in the supernatant and particulate fractions or on their relation to the wild-type enzyme (for details, see the Methods section and legend to Fig. 2). The signal of total SDS-soluble CBS antigen of the mutant proteins was generally somehow decreased with a median of 66% of the wild-type CBS (see Supp. Table S2; gels used for quantification are not shown), although some variant proteins were present in increased quantities. These data suggest that compared to the wild-type enzyme the majority of mutant CBS proteins are less stable in *E. coli*; however, we cannot exclude that some of them are also less soluble in 3% SDS.

Proportion of the SDS-soluble CBS antigen, which is present in the particulate fraction, indicates the propensity of the mutant protein to form high molecular weight aggregates and inclusion bodies. The particulate fraction of the wild-type CBS lysates contained only 12% of antigen of the total SDS-soluble signal, while more of the signal in the pellet was observed for the mutant proteins (median value was 18% of the total SDS-soluble antigen, with a range of 5 to 50%; see Supp. Table S2). These data lend support to our hypothesis that the mutant proteins in this study were in general more prone to formation of large molecular weight aggregates and/or inclusion bodies. This part of our study further indicates that the previously reported analyses of mutants CBS in the supernatants of centrifuged bacterial extracts [Kozich et al., 1993] may have underestimated the steady-state amounts of mutant proteins present in bacteria, and that extraction by 3% SDS is needed to recover the insoluble aggregated molecular species.

### Impaired Quaternary Structure of Mutant Proteins as a Measure of Their Misfolding

The published data strongly support the hypothesis that CBS deficiency may be a conformational disease caused by misfolding of mutant enzymes. Detailed studies of folding, however, require protein purification, which would be especially difficult to accomplish for the most severely affected CBS enzymes. Because misfolded polypeptide chains cannot be correctly assembled and tend to form aggregates, we examined the quaternary structure of the mutant proteins in the nonparticulate fraction of crude bacterial extracts as a surrogate marker of their folding status. To assess the oligomeric structure of the mutant CBS enzymes we used electrophoresis in gradient polyacrylamide gels under nondenaturing conditions followed by Western blotting.

Visual inspection of blots showed variable amounts of fuzzy and putatively misassembled CBS, and largely differing amounts of clearly demarcated CBS tetramers or higher order oligomers as shown in Figure 2. Subsequent quantitative analysis of the blots revealed that 88% of the total water-soluble antigen of the wild-type CBS is present as tetramers/oligomers. In contrast, these correctly assembled fractions were substantially decreased in our set of mutant proteins (median tetrameric/oligomeric signal was only 31% of the total water-soluble CBS antigen, range 1–92%; data are given in Supp. Table S2). Because the CBS antigen in the nonparticulate fraction of mutant proteins was also decreased to a median 60% compared to the wild-type, the resulting net yield of tetramers/oligomers of the mutant proteins was severely reduced (median 12% of tetramers compared to the wild-type CBS). There was no clear correlation between the location of mutant proteins in different domains and their misfolding with the exception of mutant proteins in the carboxyterminal domain that were mostly assembled correctly. In summary, the above analyses demonstrated that a substantial proportion of CBS mutant proteins formed severely reduced amounts of correctly assembled tetramers; these findings add further support to the notion that misfolding is an important pathogenic mechanism in CBS deficiency.

### Different Mobility of CBS Mutant Proteins in Native Westerns

The above-mentioned Western blot analysis revealed slight but consistent differences in migration distance of the putative tetramers/oligomers of many mutant proteins in comparison to the wild-type CBS tetramer (see Fig. 2). These mobility alterations may have resulted from charge differences, conformational changes of tetramers, or even different number of subunits in the differently migrating fractions. To explore the latter hypothesis we used the Ferguson plot to determine the molecular weight of four fractions seen for wild-type enzyme, and the molecular weight of fast migrating (K102N, S466L), slow migrating (E302K, G305R, and G307S) and fast/slow migrating (R125Q) mutant proteins. For results see Supp. Figure S1.

The apparent molecular weights of sharply demarcated and thus presumably correctly assembled oligomer fractions seen for the wild-type enzyme were 177, 398, 512, and 724kDa. Considering the limitations of the technique employing crude extracts with subsequent Western blotting and keeping in mind that CBS is known to form tetramers, the results suggest that in our electrophoretic system the wild-type CBS is present predominantly in the form of tetramers, and in small amounts also as octamers, dodecamers, and hexadecamers. Two fractions of the R125Q mutant protein were predicted to be composed of tetramers and octamers, respectively. There was only a small difference in slopes, and thus in estimated molecular weights of the remaining fast (162 and 166 kDa) and slow (199–213 kDa) migrating mutant proteins as demonstrated in Supp. Figure S1. Consequently, the estimated number of CBS subunits ranged between 2.7 and 3.5 per fraction, which is congruent with tetrameric composition of these fractions. In summary, the results of Ferguson plot do not support a hypothesis that different mobility of mutant proteins compared to wild-type CBS is caused by changes in the number of enzyme subunits. Thus, the observed varying mobility of CBS mutant proteins results most likely from subtle changes in the shape and/or charge of the assembled tetramers.

### Catalytic Activity of Mutant Proteins

To enable measurements of low residual activities of mutant proteins we first developed a sensitive method for the analysis of the CBS reaction product—cystathionine. The radioactive substrate used in previous studies, ^14^C-labeled serine, was replaced by 2,3,3- ^2^H-labeled serine, and the amount of 3,3- ^2^H-labeled cystathionine produced was determined using LC-MS/MS (Krijt et al., unpublished). Using this technique, we were able to measure reproducibly activities as low as 0.3 nmol cystathionine/hr/mg of protein, which represents about 0.2% of the average wild-type CBS activity in *E. coli* extracts.

Using the sensitive new assay we observed an extreme variation of activities ranging from 0 to 245% of the wild-type enzyme with a median activity of 3% of the wild-type CBS (for details, see Table 2). Only three mutant proteins—p.G305R, p.T262R, and p.N228K—did not exhibit any measurable residual activity above 0.3 nmol/mg/hr, whereas the remaining 24 mutant proteins had above-threshold activity in at least two of the three independent expression experiments. The activities determined in the present study are for the majority of mutant proteins congruent with the previously published values listed in Table 1. In contrast to effects on folding location of the mutant residues appears to play a more important role in affecting their catalytic activity of variant proteins. There was a tendency for mutations located in the active site, heme binding pocket, and elsewhere in the active core to exhibit rather low activities while mutations inside or on the surface of the first CBS domain and at the dimer–dimer interface were in many cases as active as the wild-type enzyme (for details, see Table 2). These data suggest that mutations in different domains of the enzyme may have substantially variable impact on its catalytic activity, and that they may not affect folding and activity simultaneously.

**Table 2:** Activities of Mutant Enzymes in Nonparticulate Water-Soluble Fractions of Bacterial Extracts

			Activity [nmol cystathionine/mg protein/hr]					
			
			Expression at 37°C			Expression at 18°C		
				
	Solvent exposure	Mutation	No addition	AdoMet (0.5 mM)	AdoHcy (0.5mM)	No addition	AdoMet (0.5 mM)	AdoHcy (0.5mM)
Active site	B	p.G148R	0.2±0.1	0.2±0.2	0.3±0.3	0.4±0.1	N/A	N/A
	B	p.G305R	n.d.	n.d.	n.d.	0.4±0.02	N/A	N/A
	B	p.G307S	0.3±0.02	0.2±0.1	0.4±0.01	0.5±0.2	N/A	N/A
Heme binding pocket	S	p.H65R	5.6±1.9	4.4±1.6	1.9±0.7	0.8±0.2	N/A	N/A
	B	p.T262R	n.d.	n.d.	n.d.	0.2±0.2	N/A	N/A
	B	p.R266K	25.3±4.2	82.3±23.0	29.4±0.03	47.8±24.7	325.3±144.7	N/A
Other locations in active core	B	p.E144K	0.6±0.4	0.9±0.6	0.5±0.5	3.3±2.8	N/A	N/A
	B	p.C165Y	1.1±0.1	0.6±0.04	0.9±0.01	0.5±0.05	N/A	N/A
	B	p.N228K	n.d.	n.d.	n.d.	0.4±0.005	N/A	N/A
	B	p.I278T	0.4±0.04	0.6±0.1	0.4±0.1	0.9±0.2	N/A	N/A
	S	p.P49L	147.9±38.9	512.8±147.3	170.9±34.1	103.4±43.2	469.4±146.0	89.8±20.7
	S	p.K102N	8.8±1.7	25.7±4.3	9.2±0.9	13.6±10.2	58.2±44.5	12.0±5.6
	S	p.R125Q	2.3±0.3	8.1±2.5	2.7±0.3	19.4±8.8	141.8±78.0	N/A
	S	p.T191M	0.4±0.02	0.2±0.2	n.d.	0.6±0.03	N/A	N/A
	S	p.E302K	136.2±19.3	147.5±10.1	112.3±4.3	81.5±28.9	95.3±29.2	107.7±68.1
	S	p.R369C	2.5±0.4	6.4±0.3	2.4±0.1	13.1±0.4	57.5±8.1	N/A
Dimer–dimer interface	S	p.P78R	13.8±5.0	43.4±12.2	13.2±4.9	26.4±9.2	149.7±36.1	31.0±8.4
	S	p.A114V	109.7±10.5	303.3±36.7	117.0±19.0	13.0±1.2	28.0±9.4	11.5±2.2
	S	p.E176K	4.9±0.6	2.7±0.3	4.4±0.6	11.5±8.3	7.9±5.6	N/A
	S	p.V180A	13.1±3.5	24.5±5.3	21.7±0.05	127.3±15.2	457.5±65.2	N/A
Connection loop between active core and regulatory domain	B	p.W409_G453del	1.2±0.2	1.2±0.2	1.4±0.1	0.8±0.3	N/A	N/A
Regulatory domain – first CBS domain	B	p.P422L	65.5±12.6	165.8±38.1	67.0±16.6	113.2±77.9	228.4±80.3	119.3±92.0
	S	p.I435T	196.5±48.2	268.872±.9	186.6±52.8	215.4±72.4	575.2±206.5	215.5±50.7
	S	p.R439Q	167.3±28.4	774.0±56.4	179.8±0.04	76.4±14.8	705.3±257.0	N/A
	S	p.D444N	233.7±39.9	817.9±101.5	313.6±22.6	117.0±27.4	675.0±174.2	140.4±3.8
	B	p.S466L	346.2±69.1	325.2±63.1	353.7±59.9	208.7±98.0	254.4±116.9	217.2±88.3
Regulatory domain – second CBS domain	B	p.L539S	1.2±0.1	1.2±0.1	1.6±0.2	4.1±0.2	N/A	N/A
Wild-type CBS			142.7±27.2	530.5±70.0	191.4±4.5	85.5±27.0	498.2±204.4	N/A
Median of all mutations (*n* = 27)			4.9	6.4	2.7	13		
Median of buried mutations (*n* = 13)			0.6	0.6	0.5	0.8		
Median of solvent exposed mutations (*n* = 14)			13.5	34.6	17.5	22.9		

Each mutant was expressed at 37 and 18°C in three and two independent experiments, respectively. The numbers in the table represent means and standard deviations of activities determined by LC-MS/MS method with the exception of three bottom lines, which show medians of groups. The effect of AdoMet and AdoHcy on activities of mutants expressed at 37°C was determined in at least 2 experiments, effect on mutants expressed at 18°C was examined only in selected cases. n.d., not detected (activity lower than the limit of quantifications, i.e. 0.3 nmol cystathionine/mg protein/hour); B, buried mutations; S, solvent exposed mutations; N/A, not analyzed.

### Effect of AdoMet and AdoHcy on the Catalytic Activity of Mutant Proteins

To get an insight into the possible pathophysiological and therapeutic implications of AdoHcy and AdoMet in CBS deficiency we studied the effect of these two compounds on the activity of mutant proteins in nonparticulate fractions of bacterial extracts. Blood concentration of AdoHcy is increased in patients with CBS deficiency [Orendac et al., 2004] due to its reverse synthesis from homocysteine and adenosine with the help of AdoHcy hydrolase, an enzyme that can be in vitro blocked with different inhibitors. AdoMet is an allosteric activator of CBS that can possibly stimulate the residual activity of mutant proteins and that can be administered as a drug in the form of tosylate.

Activity of the wild-type CBS enzyme increased 3.9 × and 1.3 × in the presence of 0.5 mM AdoMet and 0.5 mM AdoHcy, respectively (see Table 2). Because activation of CBS by AdoHcy has not yet been reported, we explored whether it could not have been caused by contamination of this compound by AdoMet. However, LC-MS/MS analysis showed that the AdoHcy standard contained less than 1% of AdoMet, indicating that the 1.3 × increase in wild-type CBS activity in crude bacterial extracts can be attributed to AdoHcy itself.

We classified without formal statistical testing the response of mutant proteins to AdoMet and AdoHcy in this simple screening system as follows: clear activation similar to the wild-type enzyme, clear inhibition, or absence of expected activation. About one-half of the mutant enzymes in the panel exhibited an unusual response to either one of the adenosyl compounds: the p.E176K was inhibited by AdoMet, the p.E302K was inhibited by AdoHcy, and the p.H65R, p.T191M and p.C165Y appeared to be inhibited by both adenosyl compounds. In addition, mutations p.E302K, p.W409_G453del, p.I435T, p.L539S, p.E144K, p.G148R, p.G307S, p.I278T, and p.S466L were not activated by AdoMet to an extent that was usually observed for the wild type CBS (see Table 2). These data show that responses of about half of mutant proteins to AdoMet and AdoHcy may differ substantially from those of the wild-type CBS. The relevance of these findings for regulation of the activity of mutant proteins and possibly for treatment of CBS deficiency is further explored in the Discussion section.

### Effect of Low Temperature on Properties of Mutant Proteins

Low temperature during expression has been shown to enhance folding of various mutant proteins that are prone to misfolding. To examine whether any of the CBS mutations under study may be rescued by more folding-permissive conditions we expressed all 27 mutant proteins at 18°C. Compared to expression at 37°C the lower temperature has not dramatically changed the total amount of SDS-soluble mutant CBS enzymes and only slightly decreased the proportion of signal in the particulate fraction (median of 18% at 37°C vs. 15% of total signal at 18°C; see Supp. Tables S2 and S3). However, the median amount of correctly folded oligomers in the water-soluble nonparticulate fraction detected on native-PAGE increased about two times from 31% of total signal at 37°C to 68% of total signal at 18°C (Supp. Table S2 and S3). Several mutant proteins responded to low expression temperature with a dramatic (i.e., more than approximately threefold) increase of the proportion of tetramers compared to the proportion of tetramers/oligomers of the wild-type CBS, namely, the p.R266K, p.R369C, p.R125Q, p.P78R, p.V180A, and p.I435T. However, this folding promoting effect of low temperature was not universal and some mutant proteins produced conversely less tetramer. All these observations indicate that for some mutant proteins more folding-permissive conditions facilitate the attainment of correct tertiary structure, which subsequently increases the assembly of tetramers.

In addition, the low expression temperature increased the median catalytic activity of mutant proteins from 3% of wild type at 37°C to a median of 13% of wild type at 18°C (see Table 2). The mutant proteins p.R266K, p.R125Q, p.R369C, p.V180A, and p.P78R exhibited more than approximately twofold increase in activity relative to the wild type, which correlated well with the increased amount of tetramer. These five mutant proteins may be considered true folding mutants as their residual activity directly correlates with the amount of tetramer. In summary, the lower expression temperature substantially increased activity of five mutant proteins, further supporting the hypothesis that deficient CBS activity may be in some instances caused by misfolding that may be alleviated by folding permissive conditions.

### Solvent Exposure of Mutant Residues as a Determinant of Folding and Activity

We hypothesized that topology of mutations in respect to solvent exposure/buriedness may have an impact on folding and assembly of mutant CBS molecules. Therefore, we compared in the next step the typical properties of CBS enzymes carrying buried versus solvent-accessible mutations by comparing median values in Table 2 and in Supp. Table S2 and S3. We have observed similar stability of these two classes of mutant proteins in *E. coli* as evidenced by identical amounts of total SDS-soluble antigen. However, the propensity of buried mutations to correctly fold and assemble is impaired, as clearly demonstrated by the lower proportion of tetrameric/oligomeric fractions in the total CBS antigen in native gels with a median of only 14% of total antigen for buried mutations versus 36% for the solvent accessible ones. More importantly, the buried mutations are about 23 times less active with median activity of only 0.4% of the wild-type CBS versus 9.4% of the wild-type enzyme for solvent-exposed mutations. In addition, the buried mutations are not activated by AdoMet (the median activation of buried mutations of 1.0 × contrasts with the 2.5 × activation of solvent exposed mutation) and seem to be inhibited by AdoHcy (median activation of buried mutations is 0.8 ×, while it is 1.3 × for solvent-exposed mutations). These differences between buried and solvent-exposed mutations appear to be further augmented by the expression at 18°C. In summary, these comparisons strongly suggest that buried mutations may lead to much severe impairment of CBS structure and function than the solvent-exposed ones.

**Figure 3:**
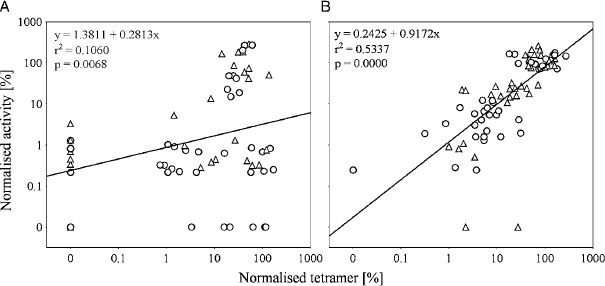
Solvent exposure and misassembly of mutants. (**A**) buried mutations; (**B**) solvent exposed mutations. Data on normalized activities and tetramer amounts shown in Supp. Figure S2 are here combined separately for all solvent exposed and all buried mutations; ^ and Δ indicate data for expression at 37 and 18°C, respectively. Linear regression analysis shows much stronger correlation between activity and the amount of tetramers for solvent exposed mutations than for the buried ones.

Expression of several mutant proteins at 18°C resulted in an increase in their catalytic activity as well as in the amount of tetramers/oligomers; we therefore proposed that these variant proteins may be considered true folding mutations. To evaluate this phenomenon in a more systematic way we have plotted the normalized activity of mutant proteins against the normalized amount of tetramers/oligomers using the combined data from five independent expression experiments. Supp. Figure S2 demonstrates that among the solvent-exposed mutations there are indeed examples of such increase of activities mirroring the increased amounts of tetramers (e.g., p.A114V, p.H65R, p.K102N, p.P78R, p.R125Q, and p.V180A). However, changes in amounts of tetramers of buried mutations were not usually accompanied by corresponding differences in activities. That led us to speculate that the solvent exposure of mutations may be indeed an important determinant of their propensity to misfold and to lose the catalytic activity. We have next performed a regression analysis in which we combined data for all buried and all solvent-exposed mutant proteins, respectively. This analysis has demonstrated that the activity of solvent-exposed mutations correlates quite strongly with the amount of oligomers (*r*^2^ = 0.53; see Fig. 3B), and suggests that for this type of mutations enhanced folding achieved by, for example, low temperature or chaperones may result in increased residual activity. In contrast, the mutations buried in the enzyme globule did not exhibit such properties, as there was only a weak correlation between the activity and the amount of oligomers (*r*^2^ = .11; see Fig. 3A). The above-described regression analysis suggests that solvent-exposed CBS mutations may be more likely to respond to interventions aimed at correcting their misfolding.

## Discussion

This article focused on testing in a systematic way the hypothesis that misfolding plays an important role in CBS deficiency. To examine the hypothesis we selected a representative set of mutants located in different functional domains of the CBS molecule. Several lines of evidence suggest that misfolding is an important pathogenic mechanism in CBS deficiency: (1) the amount of SDS-soluble mutant CBS polypeptides in a particulate fraction was increased by 50% compared to the wild type, possibly due to inclusion body formation; (2) the net yield of mutant tetramers/oligomers in the nonparticulate water-soluble fractions was severely reduced to only 12% of the wild-type enzyme; and (3) more folding permissive conditions (i.e., expression at 18°C) led to a doubling of tetramer amounts and partial restoration of activity. The mutant proteins studied in our article represent about 70% of known patient derived CBS alleles, and 18 of these 27 mutations clearly result into moderate to severe misfolding of the CBS enzyme (i.e., formation of less than 25% of tetramer of the wild type). This is an important finding that is relevant to 362 of 753 mutant alleles deposited in the CBS Mutation Database as of April 2010; in other words, about one-half of all known chromosomes affected by pathogenic mutations code for mutant polypeptides that misfold and misassemble. Taken together, all these data suggest that abnormal folding is an important and common molecular mechanism in CBS deficiency.

An important finding of our study is the more important role of solvent accessibility of mutations rather than their location in enzyme domains in predicting properties of mutant enzymes. In general, buried mutations exhibited much more profound detrimental effect on enzyme folding and activity compared to the solvent-accessible mutations. Appearance of sufficient amounts of tetramers in native gels did not predict enzyme activity for the buried mutants; however, increased tetramer formation of surface mutants elicited by low expression temperature led to an increase in residual activity. The plot of activity versus amount of tetramer (see Fig. 3 and Supp. Fig. S2) shows a good correlation for solvent-exposed mutants, implying that enzyme activity of these genetic variants is determined in part by the amount of tetramers and in part by the precise location of the mutant residue and the character of the change.

In this study we observed clearly abnormal response to AdoMet and AdoHcy for about one-half of mutant proteins: (1) several previously characterized mutants in the COOH-terminal regulatory region were hyperactive and were not further activated by AdoMet [Dawson et al., 1997; Kluijtmans et al., 1996, 1999]; these mutants were previously shown to have similar conformation to that of the activated wild-type CBS with bound AdoMet; (2) in contrast, another class of mutant proteins with low activity was either not stimulated or was even inhibited by AdoMet; and (3) several mutant proteins were inhibited by AdoHcy. It has been shown previously that whole blood or plasma concentrations of AdoHcy are grossly elevated and that the AdoMet levels are either normal or increased in patients with CBS deficiency [Heil et al., 2007; Orendac et al., 2003]. It is at present unknown whether the disturbed AdoMet and AdoHcy metabolism in CBS deficiency may modulate the residual activity of mutant enzymes and whether these observations may have any therapeutic implications.

The major limitation of our work relates to the different intracellular milieu of prokaryotic and eukaryotic cells, and especially to the differences in folding machineries and in protein surveillance/degradation systems. Therefore, the conclusions from our study have to be taken with care and have to be validated by analyses of mutant proteins that will be expressed in eukaryotic cells or obtained from tissues of animal models of CBS deficiency. Despite these limitations, utility of the simple bacterial expression systems has been appreciated for their ability to demonstrate the inherent predisposition of other mutant enzymes such as phenylalanine hydroxylase towards aberrant folding and mis-assembly [Waters, 2003].

The above-described observations may lead to the development of new strategies for treating patients with CBS deficiency. The most important implication is the potential of chaperones to rescue to a varying extent the misfolding and decreased activity of a substantial proportion of mutant proteins. We have shown previously in a selected set of mutant proteins that chemical chaperones may restore biological function of CBS mutants [Singh et al., 2007]. In addition, we examined the effect of six chemical chaperones or CBS ligands on a set of 27 mutant proteins presented in this article, and we have observed an effect of some of these compounds [Kopecka et al., in press]. However, the practical utility of chemical chaperones for the treatment of misfolding disorders is modest, and a more promising modality would be the use of a specific pharmacological CBS chaperone that awaits its discovery. The second group of treatment options implied theoretically from our present study are the AdoHcy hydrolase inhibitors that may decrease the concentrations of AdoHcy and alleviate thus inhibitory effect of AdoHcy on some mutant proteins, or the administration of AdoMet tosylate to stimulate the residual activity of other mutant CBS enzymes. AdoMet tosylate in doses of several hundreds of milligrams per day has been used in treatment of liver disease, myopathies, and depression without reports on adverse effects [Mato et al., 2001]. Whether or not chaperones and adenosyl compounds will be of any use in patients with CBS deficiency and will impove quality of their lives is presently unknown and needs to be explored in the future.
