# Supplementary material for: Cystathionine β-Synthase Mutations: Effect of Mutation Topology on Folding and Activity
Source: Hum Mutat. 2010 May 18;31(7):809–19. doi: 10.1002/humu.21273 (PMC2966864; doi:10.1002/humu.21273)
Supplement: Supplementary file 1 [file humu0031-0809-SD1.pdf]

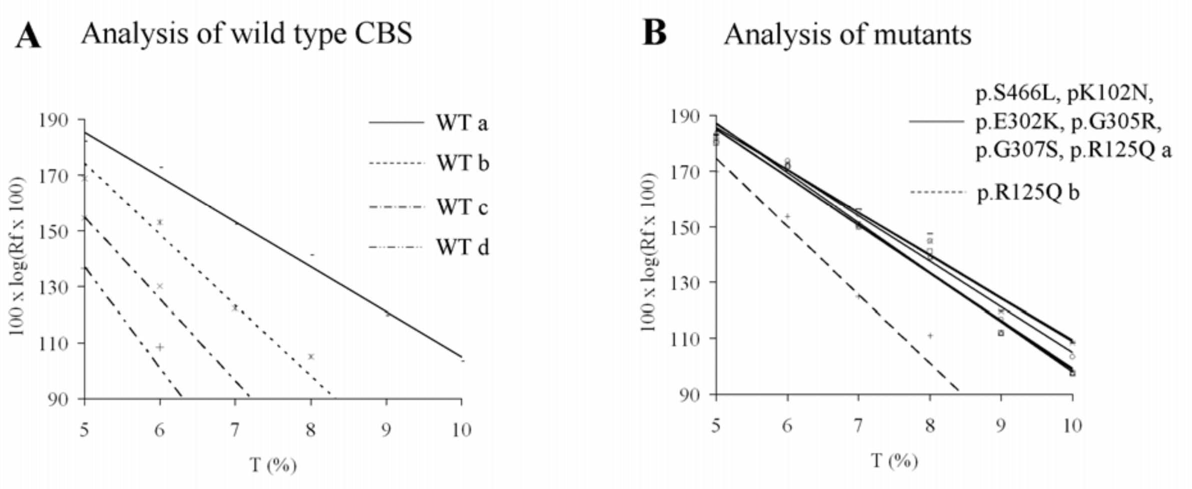

WT fractions	Negative slope	Mr	Oligomeric structure
WT a (fastest)	16.08	177	tetramer
WT b	25.39	398	octamer
WT c	29.36	512	dodecamer
WT d (slowest)	36.03	724	hexadecamer

Mutant fractions	Negative slope	Mr	Deduced oligomeric structure
p.S466L (fast)	15.383	166	tetramer
p.K102N (fast)	15.249	162	tetramer
p.E302K (slow)	17.171	199	tetramer
p.G305R (slow)	17.263	199	tetramer
p.G307S (slow)	17.849	213	tetramer
p.R125Q a (fast)	16.377	182	tetramer
p.R125Q b (slow)	24.509	372	octamer

Supp. Figure S1. Ferguson plot. The slopes of regression lines obtained by plotting the relative mobilities (R_f) of markers against the gel concentrations in logarithmic scale were used to calculate the molecular weight of CBS fractions. The tables on the bottom show the values of calculated molecular weights and the deduced oligomeric structure of the four fractions of the wild type CBS enzyme (Panel A) and of six selected mutants (Panel B).

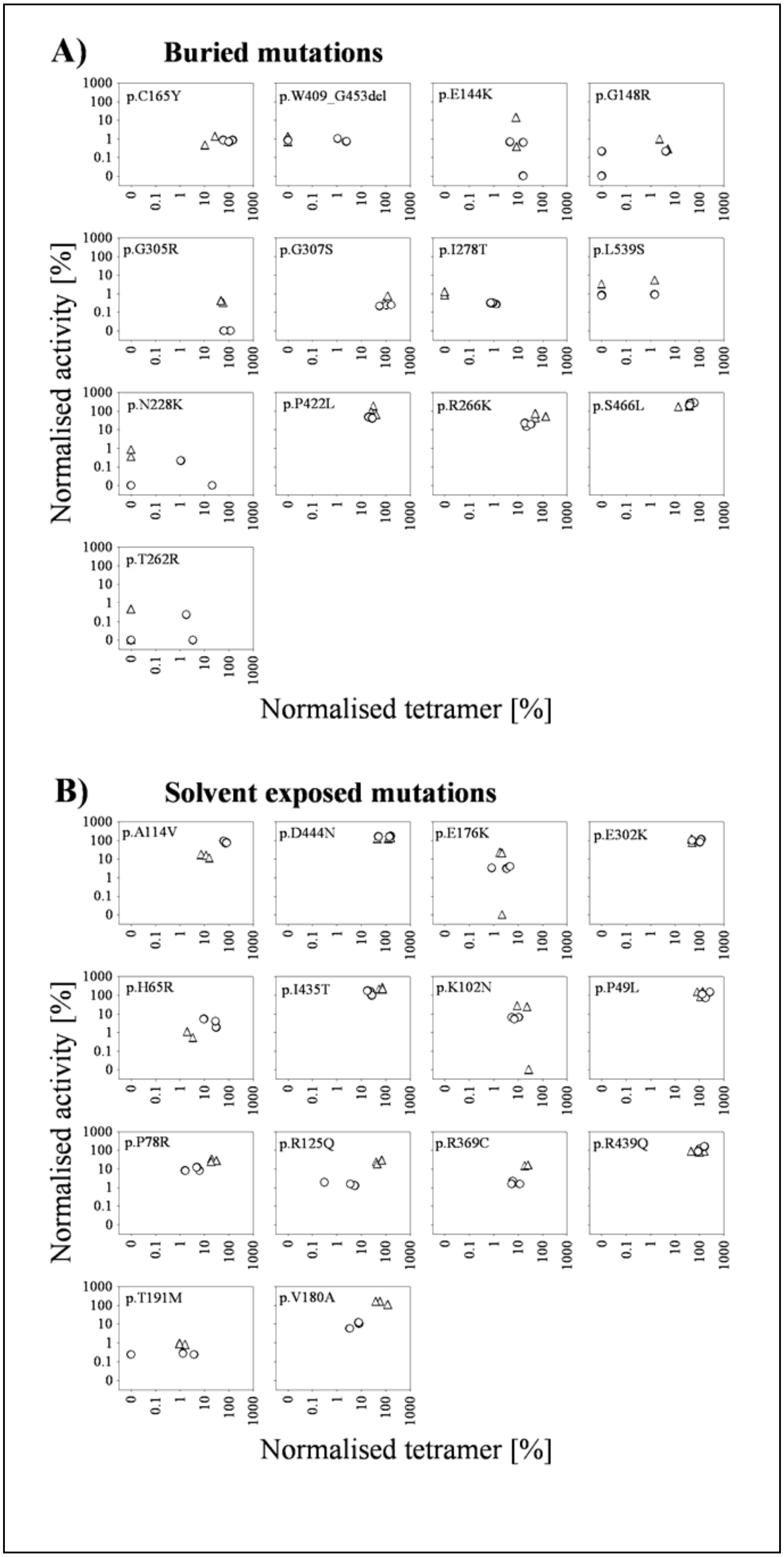

Supp. Figure S2. Relation of activity and amounts of tetramer. For each mutant data on activity in relation to the amount of tetramers/oligomers from five independent expression experiments are plotted; O and Δ indicate expression at 37°C and 18°C, respectively. All values are normalized to the amounts of tetramers and activities of the wild type CBS, data are shown on a logarithmic scale. Panel A, buried mutations; panel B, solvent exposed mutations.

Supp. Table S1. Mutagenic primers

Mutation	Primer	
p.H49L	S	5‘- gAT CCg gCC CgA TgC TCT gAg CAg gTg CAC - 3‘
	AS	5‘- gAg CAT Cgg gCC ggA TCC ACA ggg gCT CCT - 3‘
p.H65R	S	5‘- TCA CCA CAC TgC CCC ggC AAA ATC TCC AAA - 3‘
	AS	5‘- gCC ggg gCA gTg Tgg TgA CgT ggg gAC TCg gA - 3‘
p.P78R	S	5‘- AAT CTC CAA AAA TCT TgC gAg ATA TTC TgA - 3‘
	AS	5‘- gCA AgA TTT TTg gAg ATT TTg CCg ggg CAg - 3‘
p.K102N	S	5‘- gAA gAA gTT Cgg CCT gAA CTg TgA gCT CTT - 3‘
	AS	5‘- TTC Agg CCg AAC TTC TTC CCA ATC TTg TTg - 3‘
p.R125Q	S	5‘- gAT gAT TgA ggA TgC TgA gCg CgA Cgg gAC - 3‘
	AS	5‘- TCA gCA TCC TCA ATC ATC TgC Agg CTg ATg - 3‘
p.E144K	S	5‘- gCC Cgg ggA CAC gAT TAT CAA gCC gAC ATC Cg - 3‘
	AS	5‘- gAT AAT CgT gTC CCC ggg CTT CAg CgT CCC - 3‘
p.C165Y	S	5‘- AgT gAg ggg CTA TCg CTA CAT CAT CgT gAT - 3‘
	AS	5‘- AgC gAT AgC CCC TCA CTg CCg CAg CCA ggg - 3‘
p.V180A	S	5‘- CTC CgA gAA ggT ggA CgC gCT gCg ggC ACT - 3‘
	AS	5‘- CgT CCA CCT TCT Cgg AgC TCA TCT TCT CTg - 3‘
p.T191M	S	5‘- gCC CAC CAA TgC CAg gTT CgA CTC CCC ggA - 3‘
	AS	5‘- CgA ACC Tgg CAT Tgg Tgg gCA TCC TCA CAA TC - 3‘
p.N228K	S	5‘- TAC CgC AAC gCC AgC AAg CCC CTg gCT CAC - 3‘
	AS	5‘- TTg CTg gCg TTg Cgg TAC Tgg TCT Agg ATg - 3‘
p.T262R	S	5‘- CAC ggg Cgg CAC CAT CAg ggg CAT TgC CAg - 3‘
	AS	5‘- TgA Tgg TgC CgC CCg TgC CCA CTg AAg CCA - 3‘
p.R266K	S	5‘- gAA gCT gAA ggA gAA gTg TCC Tgg ATg CAg - 3‘
	AS	5‘- ACT TCT CCT TCA gCT TCT Tgg CAA TgC CCg - 3‘
p.I278T	S	5‘-TgT CCT ggA TgC Agg ATC ACT ggg gTg gAT - 3‘
	AS	5‘-TgA TCC TgC ATC CAg gAC ACT TCT CCT TCA - 3‘
p.E302K	S	5‘- Cgg AgC AgA CAA CCT ACA Agg Tgg AAg ggA - 3‘
	AS	5‘- gTA ggT TgT CTg CTC CgT CTg gTT CAg CTC - 3‘
p.G305R	S	5‘- CAA CCT ACg Agg Tgg AAA ggA TCg gCT ACg - 3‘
	AS	5‘- TTC CAC CTC gTA ggT TgT CTg CTC CgT CTg - 3‘
p.G307S	S	5‘- CTA CgA ggT ggA Agg gAT CAg CTA CgA CTT CA - 3‘
	AS	5‘- gAT CCC TTC CAC CTC gTA ggT TgT CTg CTC Cg - 3‘
p.R369C	S	5´- gCT gCg Tgg TCA TTC TgC CCg ACT CAg TgC - 3´
	AS	5´- gCA gAA TgA CCA CgC AgC ACT ggC CCT CCT - 3´
p.R439Q	S	5‘- ACA CCA TCg AgA TCC TCC Agg AgA Agg gCT TC - 3‘
	AS	5‘- ggA ggA TCT CgA Tgg TgT gCC CAC Agg Tg - 3‘
p.D444N	S	5‘- TCC ggg AgA Agg gCT TCA ACC Agg CgC CCg - 3‘
	AS	5‘- gAA gCC CTT CTC CCg gAg gAT CTC gAT ggT - 3‘
p.L539S	S	5‘- ggT CAC CgC CAT TgA CTC gCT gAA CTT CgT - 3‘
	AS	5‘- AgT CAA Tgg Cgg TgA CCA CCC CgA ACA CCA - 3‘

Sequences of mutagenic primers used to introduce the appropriate mutation into the plasmid pHCS3 which carries the wild type CBS sequence.
S, sense primer; AS, antisense primer.

Supp. Table S2. Amounts of mutant CBS antigen in fractions of bacterial extracts expressed at 37°C

	**Solvent exposure**	**Mutation**	**Temperature of expression 37°C**			
			**SDS-soluble CBS antigen in fractions**		**Water-soluble CBS antigen in supernatant**	
			**Particulate fraction of the total antigen of each mutant**	**Total antigen of mutants relative to wild type**	**Tetrameric fraction of the total mutant antigen**	**Tetramers of mutants relative to tetramer of wild type**
Active site	B	**p.G148R**	8.8 ± 2.7	52.8 ± 29.5	3.0 ± 4.3	1.4 ± 2.0
	B	**p.G305R**	7.7 ± 3.5	65.8 ± 9.1	83.4 ± 8.3	98.0 ± 24.0
	B	**p.G307S**	21.2 ± 2.3	67.9 ± 14.3	66.7 ± 9.3	111.7 ± 46.1
Heme binding pocket	S	**p.H65R**	17.7 ± 1.0	91.2 ± 12.5	31.4 ± 16.6	23.1 ± 9.5
	B	**p.T262R**	16.0 ± 5.4	49.6 ± 3.8	2.4 ± 2.3	1.7 ± 1.4
	B	**p.R266K**	21.1 ± 8.7	85.7 ± 9.3	38.4 ± 15.6	24.7 ± 6.3
Other locations in active core	B	**p.E144K**	49.6 ± 3.9	255.2 ± 165.6	4.9 ± 1.4	12.3 ± 5.4
	B	**p.C165Y**	28.8 ± 4.7	75.4 ± 41.0	68.0 ± 9.7	101.2 ± 34.7
	B	**p.N228K**	9.2 ± 3.6	37.5 ± 21.9	13.6 ± 16.7	7.4 ± 9.8
	B	**p.I278T**	28.2 ± 2.0	74.8 ± 23.9	3.5 ± 1.1	1.0 ± 0.2
	S	**p.P49L**	10.8 ± 0.3	144.9 ± 75.1	88.0 ± 1.9	194.6 ± 60.1
	S	**p.K102N**	17.6 ± 8.1	39.2 ± 4.1	29.4 ± 5.6	7.7 ± 2.3
	S	**p.R125Q**	20.5 ± 6.1	55.5 ± 22.3	7.2 ± 5.4	3.1 ± 2.1
	S	**p.T191M**	36.5 ± 10.4	35.6 ± 4.0	2.9 ± 2.2	1.7 ± 1.5
	S	**p.E302K**	11.6 ± 1.3	88.3 ± 0.1	71.2 ± 17.9	90.7 ± 27.8
	S	**p.R369C**	27.7 ± 11.5	65.8 ± 12.2	15.7 ± 6.7	7.8 ± 2.9
Dimer-dimer interface	S	**p.P78R**	17.8 ± 0.6	57.6 ± 22.3	41.3 ± 41.9	4.3 ± 1.9
	S	**p.A114V**	12.7 ± 0.4	111.1 ± 20.2	85.2 ± 3.8	73.7 ± 8.5
	S	**p.E176K**	15.0 ± 7.6	54.7 ± 3.1	17.7 ± 13.0	3.0 ± 1.6
	S	**p.V180A**	26.1 ± 1.9	54.0 ± 3.7	20.1 ± 12.7	6.6 ± 2.3

	Solvent exposure	Mutation	Temperature of expression 37°C			
			**SDS-soluble CBS antigen in fractions**		**Water-soluble CBS antigen in supernatant**	
			**Particulate fraction of the total antigen of each mutant**	**Total antigen of mutants relative to wild type**	**Tetrameric fraction of the total mutant antigen**	**Tetramers of mutants relative to tetramer of wild type**
Connection loop between active core and regulatory domain	B	**p.W409_ G453del**	23.2 ± 4.6	43.7 ± 21.7	4.3 ± 3.2	1.2 ± 1.0
Regulatory domain - first CBS domain	B	**p.P422L**	26.1 ± 11.1	76.1 ± 6.0	61.9 ± 17.5	24.5 ± 3.6
	S	**p.I435T**	20.9 ± 11.8	64.6 ± 0.4	55.2 ± 23.0	23.2 ± 4.1
	S	**p.R439Q**	10.7 ± 8.1	322.1 ± 59.6	91.6 ± 7.7	117.6 ± 29.8
	S	**p.D444N**	5.1 ± 3.0	174.6 ± 16.5	88.7 ± 5.8	120.2 ± 49.7
	B	**p.S466L**	6.4 ± 6.4	100.0 ± 49.9	67.2 ± 21.0	47.8 ± 10.2
Regulatory domain - second CBS domain	B	**p.L539S**	5.1 ± 1.9	36.6 ± 11.6	1.0 ± 1.5	0.5 ± 0.7
**Wild type CBS**			**12.4 ± 11.1**	**100.0**	**88.0 ± 10.3**	**100.0**
**Median of all mutations** (n=27)			**17.7**	**65.8**	**31.4**	**12.3**
**Median of buried mutations** (n=13)			**21.1**	**67.9**	**13.6**	**12.3**
**Median of solvent exposed mutations** (n=14)			**17.7**	**65.2**	**36.4**	**15.5**

The SDS-soluble antigen was determined by boiling the particulate and non-particulate fractions of bacterial extracts in 3% SDS and by SDS-PAGE followed by western blotting; the signal was quantified by chemiluminescence; the percentual proportion of antigen in the pellets is shown in the first column, total CBS antigen normalized to the wild type CBS is given in the second column. The ability of mutants to fold and assemble was examined by electrophoresis of water soluble non-particulate fractions under non-denaturing conditions and western blotting; percentual proportion of tetramers of the total signal is shown in the third column, yield of tetramers/oligomers in comparison to the amount of wild type tetramers is shown in the fourth column. All values with the exception of last 3 lines are means and standard deviations from 2-3 determinations.

Supp. Table S3. Amounts of mutant CBS antigen in fractions of bacterial extracts expressed at 18°C

	Solvent exposure	Mutation	Temperature of expression 18°C			
			SDS-soluble CBS antigen in fractions		Water-soluble CBS antigen in supernatant	
			Particulate fraction of the total antigen of each mutant	Total antigen of mutants relative to wild type	Tetrameric fraction of the total mutant antigen	Tetramers of mutants relative to tetramer of wild type
Active site	B	**p.G148R**	17.3 ± 14.4	23.3 ± 8.4	94.0 ± 8.4	3.2 ± 1.5
	B	**p.G305R**	9.4 ± 0.8	100.6 ± 31.7	68.4 ± 18.8	51.6 ± 7.2
	B	**p.G307S**	9.8 ± 3.9	189.1 ± 20.7	74.2 ± 9.3	97.5 ± 17.7
Heme binding pocket	S	**p.H65R**	38.0 ± 12.5	45.0 ± 7.0	12.7 ± 9.0	1.8 ± 1.4
	B	**p.T262R**	14.5 ± 8.7	28.9 ± 15.0	n.d.	n.d.
	B	**p.R266K**	27.0 ± 16.9	117.8 ± 47.5	90.6 ± 8.0	81.0 ± 40.6
Other locations in active core	B	**p.E144K**	22.5 ± 6.6	39.4 ± 28.0	41.2 ± 42.7	5.7 ± 4.0
	B	**p.C165Y**	6.6 ± 1.9	63.4 ± 8.0	56.6 ± 12.2	17.3 ± 7.0
	B	**p.N228K**	26.7 ± 7.9	59.0 ± 31.9	n.d.	n.d.
	B	**p.I278T**	43.6 ± 9.7	49.1 ± 47.0	n.d.	n.d.
	S	**p.P49L**	6.1 ± 2.3	92.0 ± 58.0	91.1 ± 2.5	112.0 ± 23.0
	S	**p.K102N**	23.1 ± 9.5	61.8 ± 12.9	64.1 ± 14.6	20.2 ± 7.8
	S	**p.R125Q**	18.0 ± 12.3	71.0 ± 1.0	84.1 ± 5.7	53.4 ± 13.0
	S	**p.T191M**	31.6 ± 14.3	23.9 ± 5.6	9.4 ± 4.6	1.1 ± 0.4
	S	**p.E302K**	15.8 ± 7.3	113.8 ± 68.5	74.0 ± 6.4	48.8 ± 1.8
	S	**p.R369C**	27.8 ± 8.4	58.5 ± 20.2	75.8 ± 8.0	23.3 ± 3.2
Dimer-dimer interface	S	**p.P78R**	4.7 ± 0.8	77.5 ± 21.6	71.6 ± 6.0	23.7 ± 5.9
	S	**p.A114V**	4.0 ± 1.4	56.5 ± 11.0	59.6 ± 17,3	12.0 ± 3.6
	S	**p.E176K**	4.9 ± 4.9	39.8 ± 7.2	32.7 ± 8.8	2.1 ± 0.2
	S	**p.V180A**	7.1 ± 0.1	136.2 ± 1.8	84.7 ± 8.9	75.0 ± 36.5

	Solvent exposure	Mutation	Temperature of expression 18°C			
			SDS-soluble CBS antigen in fractions		Water-soluble CBS antigen in supernatant	
			**Particulate fraction of the total antigen of each mutant**	**Total antigen of mutants relative to wild type**	**Tetrameric fraction of the total mutant antigen**	**Tetramers of mutants relative to tetramer of wild type**
Connection loop between active core and regulatory domain	B	**p.W409_ G453del**	22.8 ± 9.4	36.0 ± 8.6	n.d.	n.d.
Regulatory domain - first CBS domain	B	**p.P422L**	9.2 ± 8.0	78.5 ± 11.8	67.6 ± 4.0	33.3 ± 6.9
	S	**p.I435T**	15.1 ± 8.0	96.9 ± 18.6	82.7 ± 4.4	68.1 ± 10.8
	S	**p.R439Q**	14.6 ± 3.8	174.5 ± 79.1	89.0 ± 2.9	101.6 ± 45.6
	S	**p.D444N**	7.8 ± 3.3	281.0 ± 180.5	80.1 ± 7.0	113.3 ± 49.0
	B	**p.S466L**	8.3 ± 8.3	100.4 ± 61.4	74.9 ± 7.8	35.5 ± 15.9
Regulatory domain - second CBS domain	B	**p.L539S**	5.3 ± 3.0	31.7 ± 23.7	4.5 ± 3.4	0.9 ± 0.7
**Wild type CBS**			**12.7 ± 16.6**	**100.0**	**93.1 ± 3.2**	**100.0**
**Median of all mutations** (n=27)			**14.6**	**63.4**	**68.4**	**23.3**
**Median of buried mutations** (n=13)			**14.5**	**59.0**	**56.6**	**5.7**
**Median of solvent exposed mutations** (n=14)			**14.9**	**74.3**	**74.9**	**36.3**

The SDS-soluble antigen was determined by boiling the particulate and non-particulate fractions of bacterial extracts in 3% and by SDS-PAGE followed by western blotting; the signal was quantified by chemiluminescence; the percentual proportion of antigen in the pellets is shown in the first column, total CBS antigen normalized to the wild type CBS is given in the second column. The ability of mutants to fold and assemble was examined by electrophoresis of water soluble non-particulate fractions under non-denaturing conditions and western blotting; percentual proportion of tetramers of the total signal is shown in the third column, yield of tetramers/oligomers in comparison to the amount of wild type tetramers is shown in the fourth column. All values with the exception of last 3 lines are means and standard deviations from 2-3 determinations.
n.d., not detected.
